# Strategies for Developing Shape-Shifting Behaviours and Potential Applications of Poly (N-vinyl Caprolactam) Hydrogels

**DOI:** 10.3390/polym15061511

**Published:** 2023-03-18

**Authors:** Shuo Zhuo, Billy Shu Hieng Tie, Gavin Keane, Luke M. Geever

**Affiliations:** 1 Polymer, Recycling, Industrial, Sustainability and Manufacturing Center (PRISM), Technological University of the Shannon: Midlands Midwest, Dublin Road, N37 HD68 Athlone, Ireland; a00248520@student.ait.ie; 2 Centre for Industrial Service & Design, Technological University of the Shannon: Midlands Midwest, Dublin Road, N37 HD68 Athlone, Ireland; 3 Applied Polymer Technologies Gateway, Material Research Institute, Technological University of the Shannon: Midlands Midwest, Dublin Road, N37 HD68 Athlone, Ireland

**Keywords:** NVCL, temperature sensitive hydrogels, shape-shifting behaviours, actuation

## Abstract

Stimuli-responsive hydrogels are one type of smart hydrogel, which can expand/contract in water according to changes in the surrounding environment. However, it is difficult to develop flexible shapeshifting behaviours by using a single hydrogel material. This study exploited a new method to utilise single and bilayer structures to allow hydrogel-based materials to exhibit controllable shape-shifting behaviours. Although other studies have demonstrated similar transformation behaviours, this is the first report of such smart materials developed using photopolymerised N-vinyl caprolactam (NVCL)-based polymers. Our contribution provides a straightforward method in the fabrication of deformable structures. In the presence of water, the bending behaviours (vertex-to-vertex and edge-to-edge) were achieved in monolayer squares. By controlling the content and combination of the NVCL solutions with elastic resin, the bilayer strips were prepared. The expected reversible self-bending and self-helixing behaviours were achieved in specific types of samples. In addition, by limiting the expansion time of the bilayer, the layered flower samples exhibited predictable self-curving shape transformation behaviour in at least three cycles of testing. These structures displayed the capacity of self-transformation, and the value and functionality of the produced components are reflected in this paper.

## 1. Introduction

Shapeshifting of flat materials into programmed three dimensional (3D) configurations is an emerging area of research that holds a lot of promise for the development of complex materials with unprecedented functionalities and properties [1]. A wide range of shapeshifting phenomena is seen in nature. Many plants (such as pine cone [2] and mimosa pudica [3]) use the principle of cellulose orientation to guide water-driven deployment. The complex transformation behaviours are produced with more elaborate cellulose arrangements [4]. Stimuli-sensitive hydrogels are named as one type of smart or intelligent polymer, where size, shape, solubility, conductivity, permeability, viscosity, mechanics can be altered, according to changes in the surrounding environment, including temperature [5], electrical field [6], pH [7], solvent [8] and light [9]. Given the inherent water absorption/release behaviour of hydrogels, the swelling and shrinking behaviour of the smart gels have attracted a broad range of interest. Guenther et al. (2013) developed a chemical sensor taking advantage of the swelling behaviour of a functionalised pH-sensitive hydrogel [10]. However, since hydrogels are typically isotropic materials that normally undergo uniform volume swelling and shrinking under stimulation, it is difficult to achieve asymmetric shape changes such as bending, curving and buckling. This poses a challenge to achieve shape-changing behaviours using environmentally sensitive hydrogels [11].

To address this issue, researchers designed hybrid hydrogels with different compositions and network structures, which introduces a new strategy for achieving complex shapeshifting for intelligent hydrogels. The deformation occurs when force is generated. Normally, an anisotropic deformation could be made through combining two materials [12]. One is the active layer, made of stimuli-responsive materials; the other is a passive layer and is often the layer with low stiffness (for example, rigid or soft materials). Once triggered by the activation stimulus, the deformation is generated on the active layer and spurs the passive change which leads to the change in the planer dimensions and curvature [13]. The curvature of the multi-layer structures can be controlled based on the swelling ratio of the hydrogels, thickness ratio, the stiffness of the materials and the shape and size of the designs, etc. By inducing stress only in the desired directions, one additional programming parameter could be included in the form of anisotropic material properties so that more complex transformations could be achieved [14]. The multilayer structure enables accurate control in both directions and the amount of curving depends on the dimensions and material properties. However, the high shear stress levels at the interface between the layers and the complex manufacturing steps required are disadvantages of this type of structure [1].

Notably, the strategy of using a single-layer structure has also been proposed [15,16]. Instead of incorporating multiple different materials into a multilayer structure, a gradient in the material properties of a single-layer structure can be used to produce a stress gradient along the thickness of the material [1,11,12]. The gradient could be achieved by exposing the light from one side while being partially absorbed by the polymer. In addition, non-homogenous exposure of the material to the activating stimulus can also generate a stress gradient. Generally, this strategy is more suitable for shape memory polymers (SMPs). SMPs can be retained with a temporary shape without an external stimulus and return to their original shape when exposed to a suitable external stimulation, which allows SMPs to achieve the self-changing independently [17]. Compared with the multilayer structures, the high shear stress levels at the interface of two different materials can be avoided in a monolayer structure so that the risk of delamination is eliminated. However, complex manufacturing processes and limited forms of transformation limit the use of monolayers. Moreover, precisely controlling the location of the stimulus is also a huge challenge for monolayer structures [1]. Therefore, it is of great significance to develop an intelligent mono-gel that can achieve autonomous deformation.

Poly (N-vinyl caprolactam) (PNVCL) is a temperature-responsive polymer. It is well-known for its exceptional biocompatibility, solubility, thermosensitivity, and non-ionic and non-toxic properties. Furthermore, the NVCL polymer exhibits a phase transition and becomes less water-soluble as the temperature increases above the lower critical solution temperature (LCST). The LCST of the homopolymer PNVCL (32–34 °C) is close to the physiological temperature, which offers great opportunities for applications in the biomedical field, especially in targeted drug delivery systems [18]. Free radical polymerisation is a well-established method for preparing PNVCL homopolymers and copolymers [19], while photopolymerisation is a method to rapidly transform liquid monomers or macromer solution into crosslinked reliable networks, which is certainly a suitable method for the synthesis of hydrogels. However, reviewing the literature on the development of auto-deformation using temperature-sensitive polymers, nearly all the reports used Poly (N-isopropylacrylamide) (PNIPAAm) derivatives. Lee et al. (2018) created a bilayer structure that exhibited unique altered bending behaviour by combining NIPAAm with poly (vinyl alcohol) (PVA). The direction and extent of bending can be controlled by combining NIPAAm and PVA films with different swelling ratios. In addition, Lee et al. have developed a motion of grasping and releasing in response to temperature demonstrated using starfish structures [20]. Zheng et al. (2018) developed a PNIPAAm layer (featuring a lower critical solution temperature, LCST) and a poly (acrylic acid-co-acrylamide) (P(AAc-co-AAm)) layer (featuring an upper critical solution temperature, UCST), producing a reversible thermo-responsive bilayer composite structure. The actuation of this hydrogel can be operated under non-aqueous conditions [21].

4D printing is a high-precision, rapid and customizable way to prepare monolayer/bilayer smart hydrogels [22]. The mathematical modelling supports for multilateral design ensure the quality and responsiveness of the printed objects [23]. However, complicated manufacturing processes and a diversity of materials is a difficult task for an ordinary 3D printer. As a result of the high price and advanced technology of multi-layer 3D printers, they are not readily available. Therefore, the concept of constructing single/bilayer objects via UV chamber systems was proposed. The works presented in our last papers prepared a series of PNVCL copolymers/terpolymers via photopolymerisation and 3D printing [18,24]. The copolymer containing 0.1 wt% Irgacure 2959, 2 wt% poly (ethylene glycol) dimethacrylate (PEGDMA), 30 wt% N, N-dimethyl acrylamide (DMAAm) and 70 wt% NVCL demonstrated a relative short reaction time and excellent swelling behaviours. In the previous studies, as the temperature rose and fell, the reversible expansion/contraction behaviours were displayed in 4D printed jigsaw and flower architectures. However, these behaviours are limited to simple volume changes. Therefore, the main objective of this study was to use NVCL-based candidate formulations for monolayer/bilayer structures to achieve complex shape transformation behaviours. Although other researchers have developed bilayer structures and more complex deformation mechanisms, few scholars have selected NVCL-based polymers. In addition, the preparation of intelligent monolayer hydrogels is rarely reported. In this paper, a method of simply fabricating monolayer and bilayer structures is presented. In the presence of water, the shapeshifting behaviours were successfully exhibited. Additionally, depending on the transformations, the structures were used to construct a series of demonstrators.

## 2. Materials and Methods

### 2.1. Materials

N-vinyl caprolactam (NVCL) was obtained from Sigma Aldrich Ireland with a molecular weight of 139.19 g/mol and a storage temperature from 2 to 8 °C. 4-(2hydroxyethoxy) phenyl-(2-hydroxy-2-propyl) ketone (Igracure^®^ 2959 Ciba Corp, New York, United states) was obtained from Ciba Specialty Chemicals. N, N-dimethyl acrylamide (DMAAm) was purchased from Sigma Aldrich with a molecular weight of 99.13 g/mol. The chemical crosslinker used was poly (ethylene glycol) dimethacrylate (PEGDMA) supplied by Sigma Aldrich Ireland with a molecular weight of 550 g/mol.

### 2.2. Hydrogels Synthesis

The hydrogels investigated in this study were prepared by free-radical polymerisation using UV light. The chemical crosslinking hydrogels were synthesised via a UV curing system (Dr. Gröbel UV- Elektronik GmbH, Ettlingen, Germany). This particular irradiation chamber is a controlled radiation source with 20 UV-tubes that provide a spectral range between 315 and 400 nm at an average intensity of 10–13.5 mW/cm^2^. The prepolymerised mixtures were prepared by combining the predetermined amounts of the materials. The composition of the hydrogel is listed in Table 1. The batch was placed in a 100 mL beaker and mixed using a magnetic stirrer for 20 min until a homogeneous mixture was obtained. The solutions were pipetted into silicone moulds that contained designed impressions. The methods used for photopolymerisation are described in Section 2.4.1 and Section 2.4.2. All samples were dried prior to use for 24 h in a vacuum oven at 50 °C.

### 2.3. Form 2 SLA 3D Printer

Form 2 (Formlabs Company, Boston, MA, United States) is a stereolithography 3D printer developed by Formlabs. Items are constructed on the platform from bottom to top in an upside-down posture. Only files in an STL format can be accepted and uploaded to the printer. Based on a model designed in advance in computer-aided design software (CAD), the photopolymer resin is solidified by providing UV laser (405 nm) to form a single layer onto the surface of the photopolymer vat. The laser spot size of the 3D printer is 140 microns and the maximum printing size is 14.5 × 14.5 × 17.5 cm^3^, with a range from 25 to 300 microns per layer. The thickness of print for each layer is set at 50 μm.

### 2.4. Shapeshifting Designs

#### 2.4.1. Monolayer

The trials of monolayer structures were prepared in the shape of a square. The prototype of the squares was 3D printed with High temperature V2 (Formlabs Company, Boston, MA, USA) via a Form 2 3D printer (Formlabs Company, Boston, MA, USA) based on the designed dimensions of 30 mm × 30 mm × 2 mm (height × width × thickness) for silicone mould preparation.

**Figure 1:**
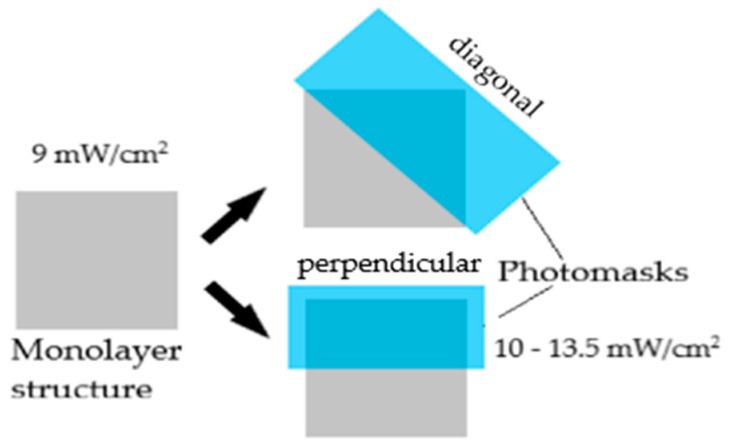
UV-curing of monolayer structures containing regions with different crosslinking degrees.

The monolayer samples were UV-cured using the Dr. Gröbel UV chamber system. The prepolymerised solution (S5) was pipetted into the square impressions of the silicone mould and cured in the UV chamber for 10 min under an intensity setting averaging 9 mW/cm^2^. Subsequently, simple photomasks made of thick card were placed on top of the partially UV-cured samples, both diagonally and perpendicularly whilst covering half of the surface area of each sample. The photomasks prepared have been verified to be fully restricting the penetration of UV wavelength using a UVP Model UVX Digital Radiometer. After that, the samples were cured for 10 min under an intensity setting averaging 10–13.5 mW/cm^2^. Figure 1 below illustrates the placements of prepared photomasks on the partially UV-cured samples.

#### 2.4.2. Bilayer

**Figure 2:**
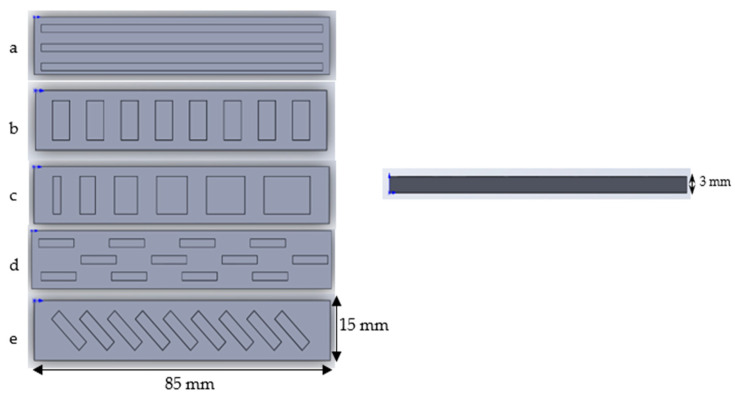
The design of the five types of bilayer strips.

**Figure 3:**
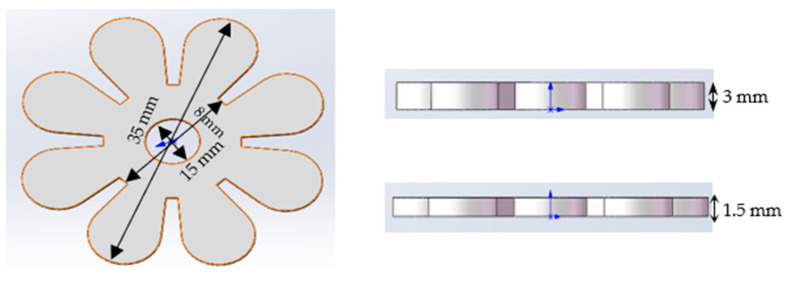
The design of the bilayer flower.

The trials of bilayer structures were prepared in the shape of rectangle strips and flowers. The prototype of the strips and flowers were 3D printed with High temperature V2 (Formlabs Company, Boston, MA, USA) via Form 2 3D printer (Formlabs Company, Boston, MA, USA) based on the design shown on Figure 2 and Figure 3 for silicone mould preparation.

The bilayers were fabricated using the Dr. Gröbel UV chamber system for the detection of self-changing behaviours. The formulation S5, which demonstrated the best performance in a previous investigation [24], was used to form the active layer. The passive layer was prepared with Elastic 50A V1 resin (Formlabs Company, Boston, MA, USA). Elastic resin is a soft and transparent photopolymer resin. 50A stands for the hardness of the material.

##### Bilayer Strips

To exploit the different shapeshifting behaviours, five styles of strips were designed. For the preparation of the strip samples, the passive layer was first generated by pipetting the elastic resin onto the shaped mould. The mould was placed into the UV chamber for 10 min to cure. After curing, the S5 solution was pipetted onto the prepared elastic shaped layer and cured for a further 10 min. All the strips are 85 mm in length, and 15 mm in width. The details of the dimensions for each strip are presented Figure 2 below.

All strips in the design are 3 mm thick.

(a) The strip consists of 3 long rectangular recesses, 2 mm in width, 81 mm in length and 1.5 mm in thickness, spaced 5 mm apart from each other.
(b) The strip consists of 8 same size rectangular recesses, 10 mm in width, 5 mm in length and 1.5 mm in thickness, spaced 5 mm apart from each other.
(c) The strip consists of 6 rectangular recesses. The length is increased by 2 mm each, from 2 mm to 12 mm. The width and thickness are maintained at 10 mm and 1.5 mm, respectively.
(d) The strip consists of 12 rectangular recesses, 2 mm in width, 10 mm in length and 1.5 mm deep arranged in three rows. Four recesses are arranged in each row 20 mm apart from each other.
(e) The strip consists of 9 identically sized rectangular recesses placed at a 45-degree angle, 11 mm in width, 3 mm in length and 1.5 mm in thickness, spaced 3 mm apart from each other.

##### Bilayer Flowers

The flower models were printed on a 3D printer at a thickness of 3 mm. In order to have the same thickness for the passive and active layers, another model with a thickness of 1.5 mm was also prepared. The two-layer flower structures were constructed by pipetting a prepared S5 solution into a 1.5 mm thick silicone mould, before being placed in the UV chamber system. In the presence of UV light, the S5 solution is cured. The cured active layer is then placed in the bottom of the 3 mm mould. After that, the elastic resin is pipetted on the top of the active layer until the 3 mm mould is full. This effectively ensures the thickness ratio between the active and passive layers. The details of the dimensions for each strip are presented in Figure 3 below.

### 2.5. Silicone Mould Preparation

**Figure 4:**
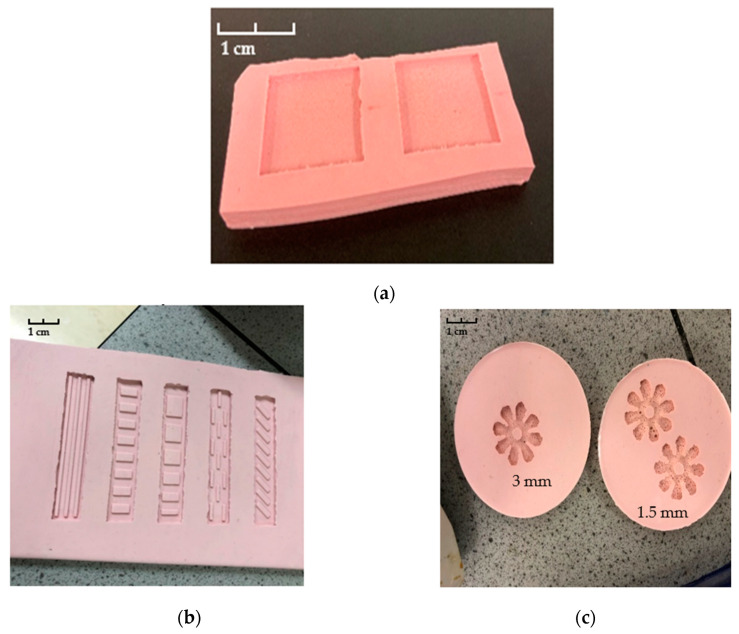
(**a**) Monolayer square silicone mould; (**b**) Strips silicone mould; (**c**) 3 mm and 1.5 mm thick flower silicone moulds.

The monolayer (square) and bilayer (strips and flower) prototypes were printed with support using an SLA Form 2 3D printer with V2 high temperature resin. The models made of high temperature resin had tough properties to avoid adhesion with silicone during the secondary processing. Then, the printed models were immersed in an IPA solution for 20 min to remove any residual resin and exposed to UV light for 30 min to guarantee the residual resin was fully cured. After that, the printed and treated prototypes were placed at the bottom of a clean glass petri dish. To prepare the silicone, the BASE and Red catalyst (Silastic 3481 and 34 series Fast Catalyst) were mixed at a ratio of 9:1 in the plastic beaker and stirred until homogeneous. Subsequently, the mixture was allowed to stand until no more bubbles were produced and then poured into the petri dish until the container was fully filled. The silicone mould was left to solidify for 24 h. The layered silicone moulds are shown in Figure 4. It can be seen that the shaped moulds were successfully established; patterns and features on all strips and flowers are clearly indicated.

### 2.6. Pulsatile Swelling Studies

The shapeshifting behaviours of the monolayer and bilayer structures was analysed by pulsatile swelling studies. The samples were placed into rectangular plastic containers of 120 × 80 cm^2^ containing 350 mL of distilled water (pH = 7.1). Samples were tested whilst switching conditions between ambient temperature and 50 °C. The plastic containers were first stored at room temperature to observe the transformations of the bilayers. Then, after a specific interval, these samples were placed into the oven to see the changes at 50 °C (above LCST). After the new transformation was reached, these plastic containers were restored to room temperature again. The test was conducted in triplicate for each sample. The shapeshifting behaviours were also recorded by taking photographs of each sample.

### 2.7. Fabrication of the Demonstrators

To explore the practicality of the layered samples, three different kinds of demonstrators were fabricated. The specific production process is as follows.

#### 2.7.1. Automatic Demonstrator

Initially, the AIT, LIT and TUS letters were designed in CAD software and uploaded to a Form 2 3D printer and printed using high temperature resin. The printed letters were then placed in the UV system for 20 mins post-cure and painted in different colours. In the meantime, the type ‘b’ bilayer strips were prepared based on the method described in Section 2.4.2. LOCTITE® 401 adhesive is a highly absorbent and water resistant. This adhesive has previously been used to bond superelastic polymers to shape memory polymers to construct bilayer structures [25]. Therefore, in order to avoid the letters falling off the strip during the experiment, they were glued to one side of the double layered strips. Subsequently, the strips with the letters were aligned neatly and fixed to the bottom of the transparent box by means of LOCTITE® 401 adhesive. Each strip was positioned 2 mm apart to ensure that the strips did not interfere with each other in the event of curving. Finally, when all the preparations had been completed, a large quantity of water was added to the container until the strips were submerged.

#### 2.7.2. Toy Train Puller

The wooden toy locomotive was purchased from Smith’s Toys (Ireland). The train head is fitted with magnets on both sides. Due to the light weight of the wooden train, two small pieces of iron were attached to the magnets to ensure that the train remained submerged throughout the experiment. A type ‘b’ double layer strip was prepared according to the method in Section 2.4.2.1. In order to clearly observe the changes that occurred, the type ‘b’ strip was coloured orange. A small hole was drilled in one end of the strip to connect the rope to the strip. The other end of the rope was tied to the chimney of the train. This created a simple connection between the train and the strip. The other end of the strip was glued to the bottom of the container with LOCTITE^®^ 401 adhesive. When all the preparations had been completed, a large quantity of water was added to the container until the train and strip were submerged.

#### 2.7.3. Gripper

Initially, the bilayer flower was prepared according to the method described in Section 2.4.2.2. Then, the bilayer flower was mounted on a long plastic burette. In order to connect the flower to the burette, a stretchable film was inserted into the burette. Thereafter, LOCTITE^®^ 401 adhesive was applied around the hole in the centre of the flower so that the flower model could be mounted on the stretchable elastic film. This ensured that the flower did not fall off during the experiment. In addition, the outlet of the burette was sealed with tape. Subsequently, the burette with the flower model was then mounted on a burette clamp. This allowed the flower to be suspended and the height of the flower to be flexibly adjusted. For this work, an automated heating water bath system was used so that the temperature can accurately be controlled over time. A thumb-sized bolt was placed at the bottom of the water bath. Finally, when all the preparations had been completed, a large quantity of water was poured into the water bath until the bolt was submerged.

## 3. Results and Discussion

### 3.1. Monolayer and Bilayer Sample Preparation

#### 3.1.1. Monolayer Preparation

**Figure 5:**
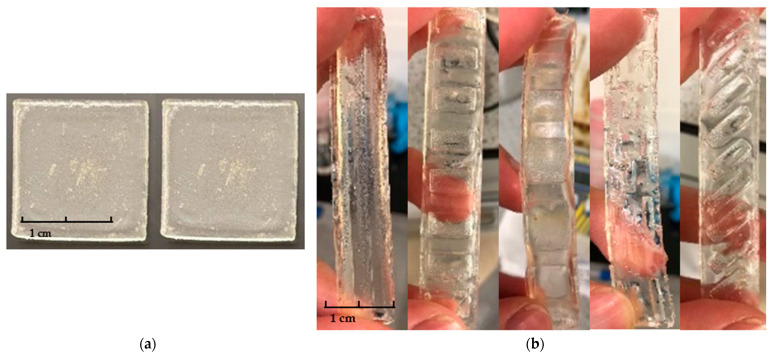
The appearance of the (**a**) monolayer squares; (**b**) bilayer strips.

As shown in Figure 5a, monolayer squares that possessed regions with different crosslinking degrees were successfully fabricated. Prior to the curing process, prepolymerised solution was filled into the silicone mould, 2 mL of solution was incorporated to ensure that each square had an equal volume. To avoid the formation of bubbles, the pipette tip was carefully positioned on one edge in each square impression when filling up the cavity. In a study conducted by Kim et al., a self-folding monolayer thin gel structure was created [26], with the technique applied in our study being more simplified. The appearance of the squares that consisted of two regions with different crosslinking degrees is indistinguishable. The surfaces of the squares were smooth and with defined edges on all corners.

#### 3.1.2. Bilayer Preparation

As shown in Figure 5b, the bilayer strips were successfully prepared. It is important to note that, in order to ensure the quality of the double layer structure, the elastic resin needs to be added to the silicone mould in several stages during UV curing. This ensures that the shape and detail of each pattern is highly reproducible. Furthermore, due to the ductility of the commercial elastic resin, the cured elastomeric resin bends to one side during the preparation. Therefore, it is necessary to use double-sided adhesive to fix the flexible mould on the platform of the UV chamber before the incorporation of the NVCL solution. All samples were successfully produced in the predefined manner. The surfaces of the strips were smooth and slightly yellow in appearance.

**Figure 6:**
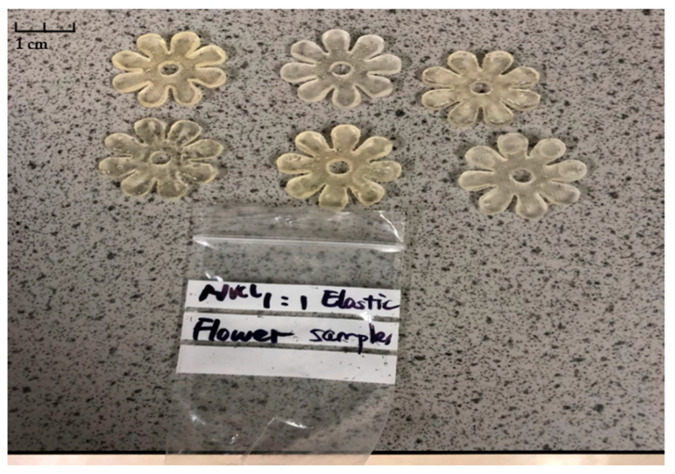
The appearances of bilayer flower samples.

In a previous study, the flower-shaped NVCL-based hydrogels were demonstrated to have excellent swelling capacity, which provided the rationale for the preparation of bilayer structures [18]. The bilayer flower structure created in this study aims to mimic the flowering process in nature. In the presence of water, the active layer will start to swell and force the passive layer to bend, thus a self-curving behaviour is expected to be achieved. The bilayer flower samples with a ratio of 1:1 between the active and passive layers were prepared and are shown in Figure 6. All flower samples were successfully prepared with flat and transparent appearances. The surface of the flower samples was smooth. In addition, the shape and integrity of the ‘petals’ were perfectly maintained.

### 3.2. Pulsatile Swelling Studies

#### 3.2.1. Monolayers

The monolayer structures made from the S5 formulation swell when being placed in the water. Hydrogels have the capability of imbibing significant amounts of fluids from the aqueous media in which they are placed, causing hydrogels to swell, while retaining the overall shape. However, a simple expansion of hydrogels should not be counted as a shape-changing mechanism, as the expanded hydrogels’ conformation is not altered although their size is enlarged. Consequently, the concept proposed in this paper is to establish different degrees of crosslinking in the monolayer hydrogel structure to allow for conformational changes in the hydrogel. Different degrees of crosslinking can be produced by manipulating the UV irradiation region [1]. These regions will have different swelling ratios, thus leading to the generation of in-plane compressive stresses upon swelling which results in buckling of the hydrogels.

**Figure 7:**
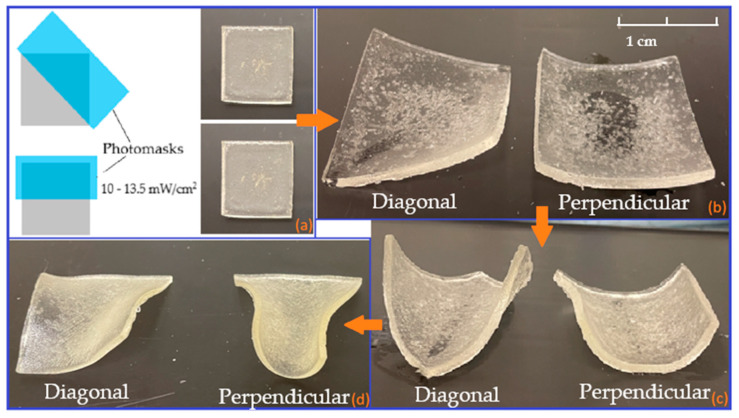
Monolayer squares swelling outcomes: (**a**) 0 h of swelling; (**b**) fully swollen state at ambient temperature after 72 h; (**c**) equilibrium state in the oven at 50 °C after 96 h; and (**d**) equilibrium state when removed from the water for drying in the oven at 50 °C after 120 h.

The swelling behaviour of monolayer squares is presented in Figure 7 below. Prior to the placement of the squares in distilled water, the samples were perfectly flat. When water was absorbed by the samples, the samples expanded as expected. However, due to each square having regions that were cured under different UV intensities and durations, the region that was irradiated with UV under higher intensity and longer duration swelled less than the other region, as different crosslinking degrees regulate the swellability of the hydrogels. Consequently, during swelling, non-uniform internal stresses were introduced within the monolayer squares that eventually led to bending of the structures. Additionally, depending on the alignments of the photomask when the squares were UV-cured at a higher intensity, both types of monolayer squares bent in different ways. The squares in which photomask was positioned diagonally also bent diagonally; and for the squares with photomask positioned perpendicularly, they bent from edge to edge.

After the fully swollen squares were moved from ambient temperature to 50 ℃, their bending became more pronounced. Unlike the study completed by Kim’s group [26], the hydrogel structures did not revert back to flat squares although being placed at a temperature that was above their LCST. This may be due to the internal stresses generated not totally being eliminated, yet the deswelling caused the degree of bending to be more noticeable as the squares became lighter when some water was released from the polymeric networks. Eventually, after the squares were fully dried in the oven at 50 ℃, the water content was completely removed from them; however, they were unable to return to their original shape. The square in Figure 7d can be seen to wrap/bend after drying, which may be due to the non-uniform internal structure.

As reported in our earlier works [18,24], a hydrogel-based monolayer only displayed volume changes under heating and cooling conversions. In contrast, a simple way of creating monolayer structures that can change their conformations was demonstrated in this contribution. In summary, the deformation responsiveness was successfully demonstrated in monolayer NVCL hydrogels. Although the shape-changing mechanism showcased was irreversible, this could, however, expand the potential of using smart shapeshifting hydrogels in specific applications and could be further finetuned to render transition reversible.

#### 3.2.2. Bilayers Strips

Another approach for achieving shapeshifting behaviour is to create a bilayer structure. The active layer of the bilayer was prepared according to the S5 formulation, with a swelling ratio of approximately 500% at room temperature in equilibrium state. Compared with the bilayers developed in other studies [24], a 500% swelling ratio is relatively large for achieving shapeshifting behaviour. This study thus proposes and practices two schemes to control the expansion of the active layer. Firstly, in a strip structure, the ratio, position and shape of the active and passive layers were altered. When a small number of active layers regularly combines with passive layers, the forces generated by water absorption and expansion are reduced, thus preventing the passive layers from being damaged in the process. In other words, the expansion capacity of the active layer is balanced by reducing the proportion of the active layer so that the expected self-deformation behaviours can be exhibited. In addition, because the direction of the force applied has a strong influence on the final shape-change behaviour, the strip models have been designed to be semi-enclosed. Secondly, at room temperature, the swelling ratio of the S5 hydrogels are increased with time. In the early stage of testing, the expansion rate of the active layer was low. Therefore, increasing the temperature in time before the hydrogel reaches equilibrium can effectively interrupt the increase in the swelling ratio, and result in the desired level of deformation.

**Figure 8:**
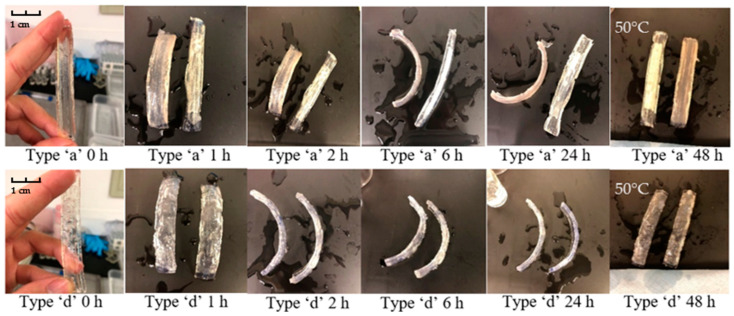
The appearance of swollen type ‘a’ and ‘d’ bilayer samples at times: 0 h; 1 h; 2 h; 6 h; 24 h and 48 h (50 °C).

The swelling tests on the double-layer structures was carried out in triplicate. The prepared strips were separately placed into large plastic trays to ensure that the samples could be fully submerged in water throughout the experiment. Almost all self-deformation behaviours developed for hydrogel-based materials exhibit a bending–unbending responsiveness [11,21,27]. The photographs in Figure 8 below were taken at different times and show the shape change behaviour of the ‘a’ and ‘d’ type samples in water. For the type ‘a’ strips, although some samples showed the expected behaviour after 24 h, consistency of the shape change was an issue. The two strips exhibited two different transformation modes. It was also noticeable that some of the active layers at the sides of the strips were damaged due to the large deformation at the ends of the strips. This may be attributed to the forces generated by the scaled active layer still being too strong, causing the strips to fold laterally rather than longitudinally. In contrast, all strips of type ‘d’ exhibited the predetermined changes. The orientation and angle of curvature of the samples at any given moment remained generally consistent. After 24 h, when the expansion of the active layer reached its maximum size, the bilayer strips showed a less noticeable angle of deformation. Additionally, when the temperature was raised above the LCST, the deformed strips failed to return to a perfectly flat formation. This is because the active layer de-swells at high temperature, but the shrunken sample still retains a 200% expansion ratio (the measurement of expansion ratio is described in reference [24]), so that only a reduction in the bending angle can be observed in the samples.

**Figure 9:**
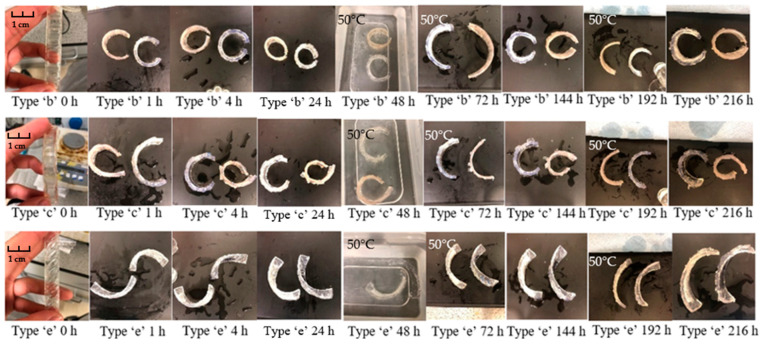
The appearances of swollen type ‘b’, ‘c’ and ‘e’ bilayer samples times: 0 h; 1 h; 4 h; 24 h; 48 h (50 °C); 72 h (50 °C); 144 h; 192 h (50 °C) and 216 h.

Comparing the type ‘a’ and ‘d’ samples, by specifying and limiting the direction and volume of the expansion, the samples of types ‘b’, ‘c’, and ‘d’ exhibited better repeatable shape-change behaviours as the temperature increased and decreased. The responsiveness of the shape changing has been analysed in at least three cycles of testing, as shown in Figure 9. The bending behaviour is reflected in the variation of the distance between the two sides of the strip. At room temperature, the type ‘b’ flat strips started to bend, resulting in a reduction of the distance between the two ends from 85 mm (length of the strip) to about 26 mm. After 6 h of immersion, the distance reduced to around 5 mm. When the active layers reached equilibrium state, the flat strips were able to curve to a loop (0–2 mm). As the temperature rose to 50 °C, the active layers shrank to a relatively small expansion size [24], thus reducing the bending angle of the bilayer strips. Due to the fact that the active layer remains in an expanded state, samples cannot fully recover to their original shape. After 48 h immersed at 50 °C (which is type ‘b’ strip shown at 72 h in Figure 9), the distance between the two sides increased to about 48 mm. After 144 h, when the temperature returned to room temperature, the increased water absorption capacity of the active layer allowed for a second increase in the bending of the bilayer strip (the distance between the two sides reduces to 2–5 mm). In the bending process, it is worth noting that the hydrogel layer did not separate from the passive layer during the entire pulsatile swelling test, which indicates the integrity and consistency of the shape change behaviour.

The rectangular design on the ‘c’ strip is progressively increasing in size. Different areas of the active layer can exert different forces on the passive layer so self-rolling behaviour was expected to be achieved in water. At room temperature, the strip started to curl due to the expansion of the active layer. However, the ‘c’ type strip failed to achieve the expected responsiveness; instead, it showed similar bending behaviour to the ‘b’ type as the water absorption increased. This may be due to the fact that the active layer does not generate enough force to produce a greater degree of curling in the strip. In the subsequent heating/cooling cycles, the strip exhibited a consistent change in the degree of bending. In the case of type ‘e’ strips, the active layer was designed at an angle of 45 degrees, which was expected to induce the bilayer strips to helix in the 45° direction. As can be seen in the images, type ‘e’ showed a different level of responsiveness than type ‘b’ and ‘c’ samples. At room temperature, though no helical structure is formed, the ‘e’ strip shows a nearly 90° folded posture when the NVCL layer is fully expanded. As the temperature increased to 50 °C, the strips reverted to a slightly bent state. The manifestation of this responsiveness offers the possibility of using such hydrogel materials to develop behaviours that are distinct from bending–unbending.

#### 3.2.3. Bilayer Flowers

**Figure 10:**
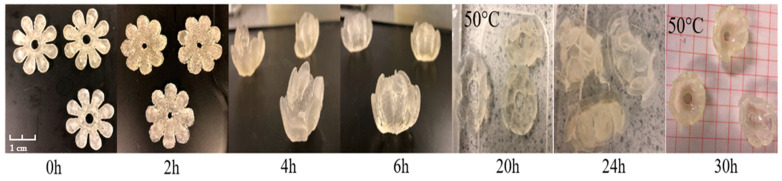
The appearance of swollen bilayer flower samples at time: 0 h, 2 h, 4 h, 6 h, 20 h (50 °C), 24 h and 30 h (50 °C).

The pulsatile swelling tests were carried out on bilayer flower samples in triplicate. The dry and flat samples were placed in a large plastic tray at 0 h to ensure there was sufficient water for deformation throughout the experiment. As demonstrated in Figure 10, at room temperature, the fully opened ‘flowers’ started to curve due to water absorption. The angle of the curve of the ‘flower’ increased as the rate of water absorption increased. After 6 h, the flower samples closed completely. The self-curving behaviour was displayed. However, as the active layer continued to swell to its equilibrium state, the passive layer was unable to withstand the curving forces generated by the active layer, resulting in damage to the sample. Therefore, the closed flowers were placed at 50 °C after 6 h, which prevented the swelling rate from increasing. This operation yielded excellent results. After 20 h, the completely closed flowers were reopened due to the contraction of the active layer. In the subsequent warming and cooling transitions, the double-layered flower samples showed the expected opening and closing process, which successfully simulated the flowering process in nature.

In summary, by combining NVCL with an elastic material, simple bilayer strip samples were produced. All samples were able to show transformation behaviour. However, these types of bilayers were difficult to apply in practice due to the following reasons: inconsistent change behaviour (type ‘a’) and inconspicuous change behaviour (type ‘d’). Only the type ‘b’ and ‘e’ strips demonstrated acceptable reproducible shapeshifting behaviours. Although the strips were unable to return to their original shape as the temperature rises, the apparent angular changes throughout the expansion tests demonstrated the intelligence and potential value of these strips. For the bilayer flower structures, by limiting the expansion time at room temperature, the flower samples prepared in this contribution exhibited predictable and reversible shapeshifting behaviour.

### 3.3. Demonstrations

The bilayer structures produced in this study show promising shapeshifting behaviours. This provides the opportunity to develop practical applications using the bilayer structures.

#### 3.3.1. Demonstration I: TUS Demonstration

**Figure 11:**
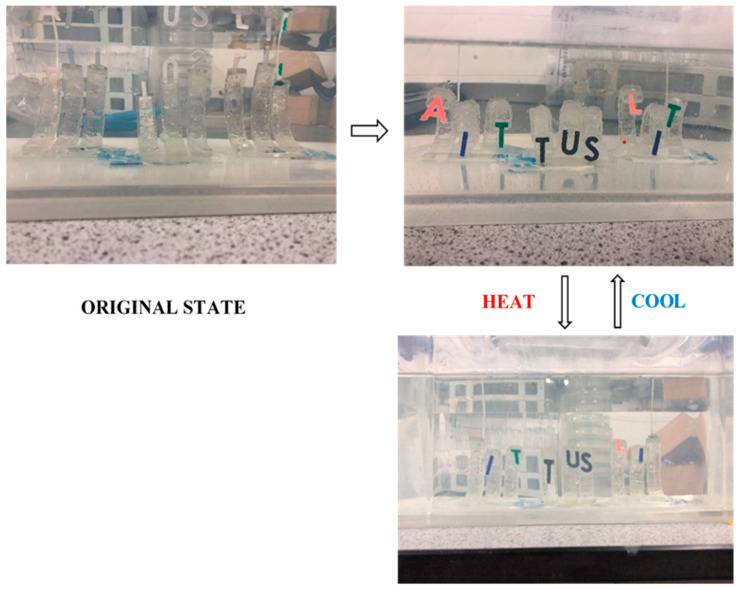
The TUS automatic displayers are achieved by using ‘b’ type bilayer strips.

As shown in Figure 11, the first application developed in this section is the TUS automatic displayer. At room temperature, it can be observed that the strips exhibit a certain degree of natural bending in water at the beginning of the swelling. This may be caused by the buoyancy of the water. After 24 h, when the active layer of the strips swelled to its maximum size, the double-layered strips underwent a self-bending behaviour, causing the “AIT”, “TUS” and “LIT” symbols to be displayed on the front of the container. When the temperature increased to 50 °C, the active layer of the strip shrunk, leading to a decrease in the expansion volume. Therefore, a decrease in the bending angle of the bilayer strip can be observed. This resulted in the three sets of letters lifting backwards and not being fully presented on the front side of the container. As the temperature returns to ambient, the “AIT”, “TUS” and “LIT” symbols are again displayed on the front side due to the increase of the water uptake. These performances marked the successful development of the automatic demonstration concept, though further finetuning could be carried out in the future to further improve consistency of the system.

#### 3.3.2. Demonstration II: Toy Train Puller

**Figure 12:**
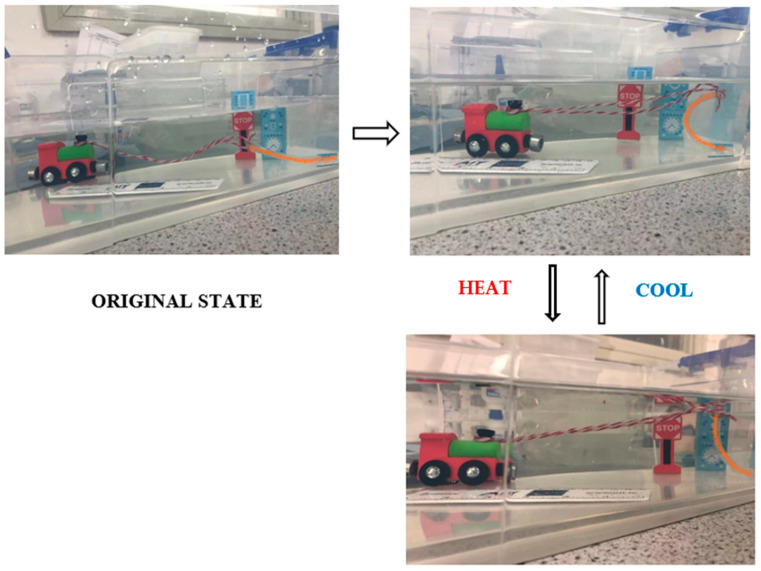
The toy train automatic puller achieved by using ‘b’ type bilayer strips.

As shown in Figure 12, the second application developed in this section is a toy train puller. At room temperature, it can be observed that the rope was loose in the absence of tension at the beginning of the swelling. The train stopped at 0 cm. As the active layer expands, the self-curving behaviour occurred, thus driving the rope to pull the train. After 24 h, the active layer expanded to its equilibrium state, which resulted in a maximum bending angle of the bilayer strip. This generated a tension force acting on the rope and propelling the train forward. At maximum expansion state, the train was pulled forward to 10 cm. These performances approved the successful development of the automatic puller.

#### 3.3.3. Demonstration III: Gripper

**Figure 13:**
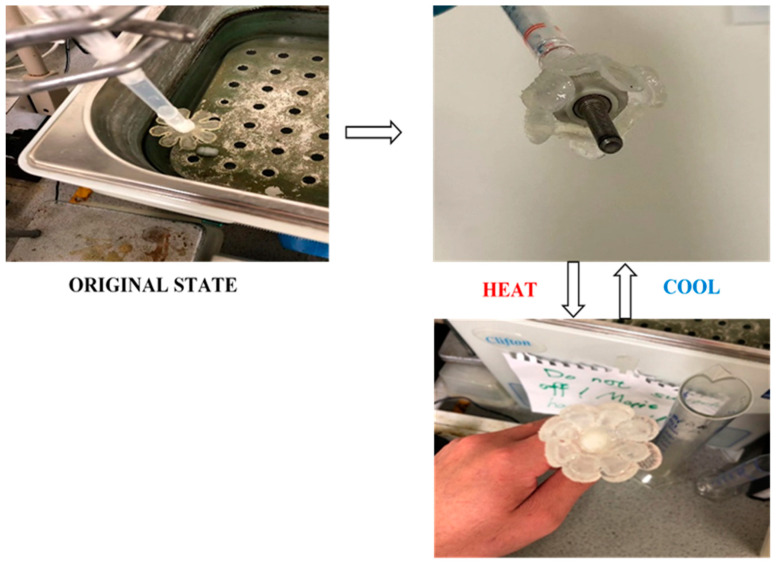
The flower gripper achieved by using bilayer flowers.

As shown in Figure 13, the third application developed in this section is a flower-shaped gripper. At the beginning of the experiment, the bilayer flower was fully opened at the top of the bolt. At room temperature, as the active layer expanded, the ‘petals’ began to bend inward and wrap around the bolt. After 6 h, it was observed that the bolt was wrapped in the petals and could easily grip the bolt. This phenomenon is attributed to the force provided by the water absorption of the active layer, which allows the petals to close completely. When the temperature was raised to 50 °C, the bolt was released. This was due to the fact that the active layer of the bilayer structure started to shrink, resulting in a reduction in the curvature of the petals. The open petals do not provide enough support to grip the bolt. When the temperature dropped back down to ambient temperature, the angle of the bilayer flower bend increased again and the bolt was again gripped.

## 4. Conclusions

In this study, shape-shifting behaviours were successfully displayed in the prepared NVCL monolayer and bilayer structures. These structures were prepared by exposing the formulated NVCL solution to different UV intensities and by adding a layer of cured PNVCL polymer on top of a commercial elastic resin via a UV curing system, respectively. As a result, the predetermined shapeshifting behaviours were demonstrated in the specific type samples. For the monolayer squares, by shading of the squares diagonally and vertically, two-corresponding bending behaviours (vertex-to-vertex and edge-to-edge) were achieved. For the bilayer strips, by regularly adding the prepared NVCL solution to the elastic strips, the type ‘b’ and ‘e’ strips demonstrated reversible self-bending and self-helixing behaviours, respectively. For the ‘flower’ structures, by limiting the expansion time of the bilayer flower at room temperature, the bilayer flowers exhibited predictable self-curving shapeshifting behaviours. Moreover, according to the achieved shape-shifting behaviours, a series of demonstrator prototypes were produced and shown to have promise. Those findings discovered in the present study showed a new method and material for self-transformation development. This not only broadens the application prospects of NVCL materials, but also provides more material options for developing self-deforming behaviours.

This article provides a rapid and potential mass-production method for preparing layered structures. It is theoretically applicable to all stimuli-sensitive hydrogels without using a 4D printer. In contrast to the 4D printing technique, it must be noted that there are some limitations and weaknesses of this approach. For example, when a new design is proposed, it requires the support of a new mould which can be time-consuming. In addition, it would not be suitable for the design of some complex three-dimensional models. That being said, the above approach still shows a lot of promise for less sophisticated shapeshifting concepts which could be produced in mass scale at relatively low cost compared to 4D printing.

## Figures and Tables

**Table 1:** The formulation of the samples used in this study.

Sample Codes	Photoinitiators	Monomers		Crosslinker
	Irgacure 2959	NVCL	DMAAm	PEGDMA
	(wt %) (365 nm)	(wt %)	(wt %)	(wt %)
S5	0.1	70	30	2

## Data Availability

Not applicable.
